# Sgf73, a subunit of SAGA complex, is required for the assembly of RITS complex in fission yeast

**DOI:** 10.1038/srep14707

**Published:** 2015-10-07

**Authors:** Xiaolong Deng, Huan Zhou, Guiping Zhang, Wenchao Wang, Langyong Mao, Xing Zhou, Yao Yu, Hong Lu

**Affiliations:** 1 State Key Laboratory of Genetic Engineering, School of Life Sciences, Fudan University; 2 Shanghai Engineering Research Center Of Industrial Microorganisms, Shanghai, China, 200438; 3 Shanghai Center for Plant Stress Biology, Shanghai Institutes for Biological Sciences, Chinese Academy of Sciences, Shanghai, China, 201602; 4 Shanghai Collaborative Innovation Center for Biomanufacturing Technology, Shanghai, China, 200237

## Abstract

RNA interference (RNAi) is a widespread gene-silencing mechanism and is required for heterochromatin assembly in a variety of organisms. The RNA-induced transcriptional silencing complex (RITS), composed of Ago1, Tas3 and Chp1, is a key component of RNAi machinery in fission yeast that connects short interference RNA (siRNA) and heterochromatin formation. However, the process by which RITS is assembled is not well understood. Here, we identified Sgf73, a subunit of the SAGA co-transcriptional complex, is required for pericentromeric heterochromatin silencing and the generation of siRNA. This novel role of Sgf73 is independent of enzymatic activities or structural integrity of SAGA. Instead, Sgf73 is physically associated with Ago1 and Chp1. The interactions among the subunits of the RITS, including those between Tas3 and Chp1, between Chp1 and Ago1, between Ago1 and Tas3, were all impaired by the deletion of *sgf73*^+^. Consistently, the recruitment of Ago1 and Chp1 to the pericentromeric region was abolished in *sgf73*Δ cells. Our study unveils a moonlighting function of a SAGA subunit. It suggests Sgf73 is a novel factor that promotes assembly of RITS and RNAi-mediated heterochromatin formation.

RNA interference (RNAi) is a gene-silencing mechanism widespread in eukaryotes. RNAi is mediated by small RNAs, including microRNA, Piwi-interacting RNAs and short interfering RNAs (siRNA). Small RNAs bind with Argonaute protein and guide the complex to complementary sequences for repression^1^. RNAi is well known to regulate post-transcriptional silencing within the cytoplasm^2^. On the other hand, pilot work in fission yeast^3^, along with parallel findings in multicellular organisms^4^,^5^,^6^,^7^, demonstrate that RNAi also triggers chromatin modifications, leading to heterochromatin assembly and transcriptional silencing.

Heterochromatin is a heavily condensed form of chromatin. In fission yeast, constitutive heterochromatin regions are found at telomeres, the silent mating-type locus, and pericentromeric repeats^8^. During S phase, *dh* and *dg* repeats from the outer centromeric region are transcribed by RNA polymerase II^9^. The transcripts are transcribed by an RNA-directed RNA polymerase complex (RDRC) into double-stranded RNA (dsRNA), and are processed into siRNAs by Dicer (Dcr1)^10^. These siRNAs pass through the Argonaute chaperone complex (ARC) and then are loaded onto the RNA-induced transcriptional silencing complex (RITS)^11^. RITS is composed of Argonaute (Ago1), Chp1 and Tas3, with Tas3 bridging Ago1 and Chp1 to form a linear architecture^12^,^13^. siRNAs guide RITS to the repeats region through base-pairing with the nascent transcripts, and the transcripts are sliced by Ago1^14^. Transcripts-bound RITS recruits RDRC to promote further dsRNA and siRNA production^15^. RITS also recruits histone methyltransferase Clr4 to initiate H3K9 methylation (H3K9me)^16^. H3K9me is bound by Chp1, which stabilizes the association between RITS and chromatin^17^. H3K9me serves as a platform to attract other heterochromatin components, including Swi6, Chp2 and SHREC complex, to compact the chromatin further^18^,^19^,^20^. As a key player in the RNAi-mediated heterochromatin assembly, RITS connects transcript cleavage, siRNA production and chromatin modifications^21^. However, the process by which RITS is assembled and regulated is still not well understood.

The Spt–Ada–Gcn5–acetyltransferase complex (SAGA) is a highly conserved transcriptional co-activator, which is implicated in transcriptional initiation, elongation and mRNA export. Subunits of SAGA can be assembled into modules with distinct activities. These include the structural module (Spt7, Ada1, Spt20), histone acetyltransferase (HAT) module (Gcn5, Ada2, Ada3, Sgf29), and the histone deubiquitylation (DUB) module (Ubp8, Sgf11, Sus1, Sgf73)^22^,^23^. Sgf73 serves to anchor the DUB module to SAGA and thus to fully activate the catalytic activity of Ubp8^24^. In addition, Sgf73 is involved in the establishment of a heterochromatin boundary to block the spread of silencing in budding yeast, and this requires the HAT activity of SAGA^25^. Here, we show that Sg73 is required for siRNA production and heterochromatin silencing in fission yeast. Unexpectedly, this novel role of Sgf73 is independent of the enzymatic activity or the structural integrity of SAGA. Instead, Sgf73 is physically associated with RITS. Notably, Sgf73 is required for the integrity of RITS and its recruitment to the heterochromatin region. The results suggest Sgf73-mediated assembly of RITS is critical for RNAi-dependent silencing.

## Results and Discussion

### Sgf73 is required for heterochromatin silencing

**Figure 1:**
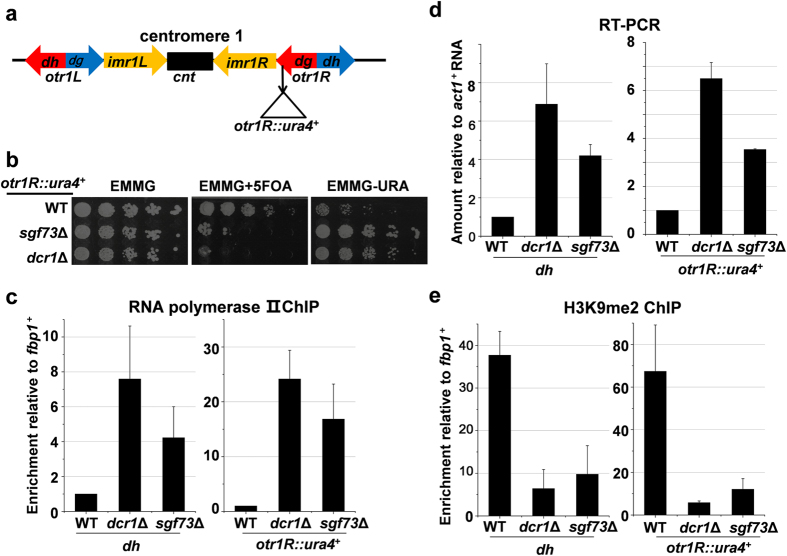
Sgf73 is required for heterochromatin silencing at pericentromeric region. (**a**) A schematic representation of centromere 1 and the position of a inserted marker gene (*otr1R*::*ura4*^+^). (**b**) Fivefold serial dilution assay to examine the silencing of *otr1R*::*ura4*^+^. Wild-type (WT) cells with silenced *ura4*^+^ grow normally on medium containing 5FOA, while loss of silencing kills cells on 5FOA. (**c**) RT-PCR analysis of *otr1R*::*ura4*^+^ and pericentromeric repeat (*dh*) RNA levels relative to a control *act1*^+^. The relative level in WT cells was arbitrarily designated as 1. Each column shown in (**c**) and below represents the mean ± s.d. from three biological repeats. (**d**,**e**) ChIP analysis of enrichment of Pol II (**d**) and H3K9me2 (**e**) at *otr1R*::*ura4*^+^ and *dh* relative to *fbp1*^+^. Relative enrichment in WT cells was arbitrarily designated as 1.

Our previous study showed that *sgf73*Δ cells are sensitive to thiabendazole (TBZ), a microtubule destabilizing drug^26^. Hypersensitivity to TBZ could arise from defects in centromeric heterochromatin, because heterochromatin is required to attract cohesin and ensure proper chromosome segregation^27^. To validate the potential role of Sgf73 in heterochromatin organization, we used a strain in which a *ura4*^+^ marker gene was inserted into the outermost (otr) pericentromeric heterochromatin of chromosome 1 (*otr1R*::*ura4*^+^) (Fig. 1a)^28^. Just as with cells lacking an essential effector of the RNAi machinery (*dcr1*Δ), deletion of *sgf73*^+^ (*sgf73*Δ) derepressed the *ura4*^+^ gene, resulting in poor growth on medium containing counterselective drug 5-fluoroorotic acid (5FOA) and good growth on medium without uracil (Fig. 1b). Consistently, the transcripts from the *otr1R*::*ura4*^+^ and endogenous pericentromeric repeat (*dh*), and the occupancy of RNA polymerase II at both loci increased substantially in *sgf73*Δ cells, indicating impaired heterochromatin silencing at the pericentromeric region (Fig. 1c,d). A hallmark of heterochromatin is the presence of histone H3K9 methylation^29^. Like *dcr1*Δ cells, *sgf73*Δ cells exhibited reduced levels of H3K9 dimethylation (H3K9me2) at centromeric repeats (*dh*) and at the inserted marker *ura4*^+^ (Fig. 1e). Thus, we conclude that *sgf73*^+^ is essential for maintaining heterochromatin silencing and repressive histone modifications at the pericentromeric region.

### Sgf73 mediates heterochromatin silencing in a SAGA-independent manner

Sgf73, along with the subunits of the HAT module of the SAGA complex, including Gcn5, Ada2, Ada3 and Sgf29, were shown to act as anti-silencing factors to prevent the spreading of heterochromatin in budding yeast^25^,^30^,^31^,^32^,^33^. Unexpectedly, Sgf73 was found to promote silencing in fission yeast in this study (Fig. 1). Therefore, it was of interest to investigate whether the role of Sgf73 in heterochromatin silencing was mediated in the context of the SAGA complex.

**Figure 2:**
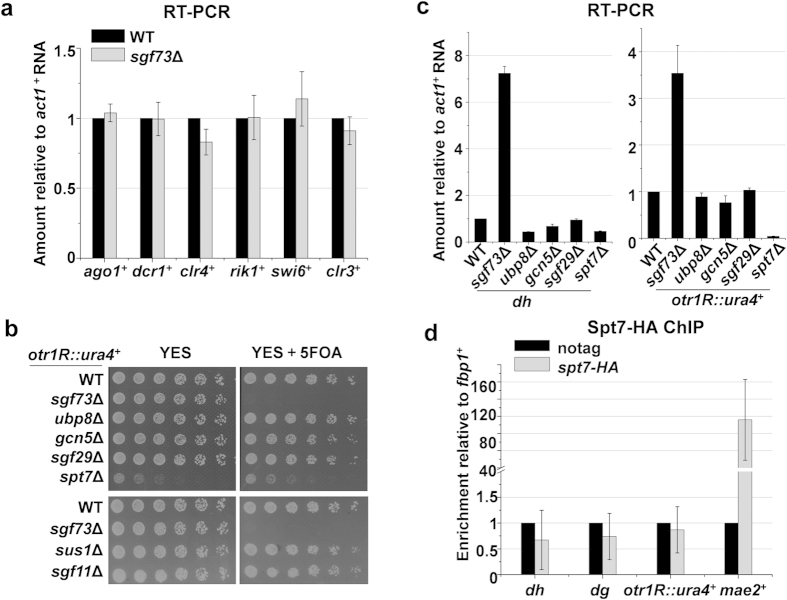
The role of Sgf73 in heterochromatin silencing is independent of enzymatic activity and structural integrity of SAGA complex. (**a**) RT-PCR analysis of RNA levels of representative factors essential for the heterochromatic silencing. The relative level to a control *act1*^+^ in WT cells was arbitrarily designated as 1. Each column shown in (**a**) and below represents the mean ± s.d. from three biological repeats. (**b**) Fivefold serial dilution assay to examine the silencing of *otr1R*::*ura4*^+^ in deletion mutants of representative SAGA subunits. (**c**) RT-PCR analysis of *otr1R*::*ura4*^+^ and pericentromeric repeat (*dh*) RNA levels relative to a control *act1*^+^. (**d**) ChIP analysis of enrichment of Spt7-3HA at *dh*, *dg*, *otr1R*::*ura4*^+^ and *mae2*^+^ relative to *fbp1*^+^. Relative enrichment in the cells without tagging (no tag) was arbitrarily designated as 1.

SAGA is responsible for the transcription of a broad range of genes. For example, 10% of genes in budding yeast are subjected to regulation by SAGA upon stress response^34^. However, the mRNA levels of several factors critical for the heterochromatin assembly in *sgf73*Δ cells were not substantially different with those in WT cells (Fig. 2a, Supplementary Fig. S1). The factors subjected to assay include Ago1, Dcr1, Clr4, Rik1, Swi6, Clr3, Arb1, Arb2, Chp1, Cid12, Raf1, Stc1, Hrr1, Rdp1, Tas3, Clr1, Swi6 and Dsh1. These data are consistent with a previous microarray analysis^35^. They suggest that Sgf73 regulates heterochromatin silencing without affecting the transcriptions of these essential factors.

To identify the contributions of SAGA to heterochromatin silencing, representative subunits of SAGA were deleted in a strain carrying an *otr1R*::*ura4*^+^ reporter system. In contrast to a severe silencing defect observed in *sgf73*Δ cells, deletion of other subunit of the UBP module, including Sus1, Sgf11 or the catalytic subunit Ubp8, did not affect silencing of *ura4*^+^ (Fig. 2b). Similarly, deletion of subunits of the HAT module, including Sgf29 or the catalytic subunit Gnc5, exhibited no silencing defect (Fig. 2b). Spt7 is a pivotal structural subunit of the SAGA complex. Deletion of Spt7 causes the dissociation of most modules and thus abolishes the activity of the SAGA complex^36^. As shown in Fig. 2b, albeit suffering a severe growth defect, *spt7*Δ cells maintained normal silencing of *ura4*^+^. Consistently, transcript levels of *dh* and *otr1R*::*ura4*^+^ in the selected SAGA subunit mutants were kept low as in WT cells, except in the *sgf73*Δ cells (Fig. 2c). Therefore, these results suggest enzymatic activities and structural integrity of SAGA are not required for the heterochromatin silencing at pericentromeric region.

**Figure 3:**
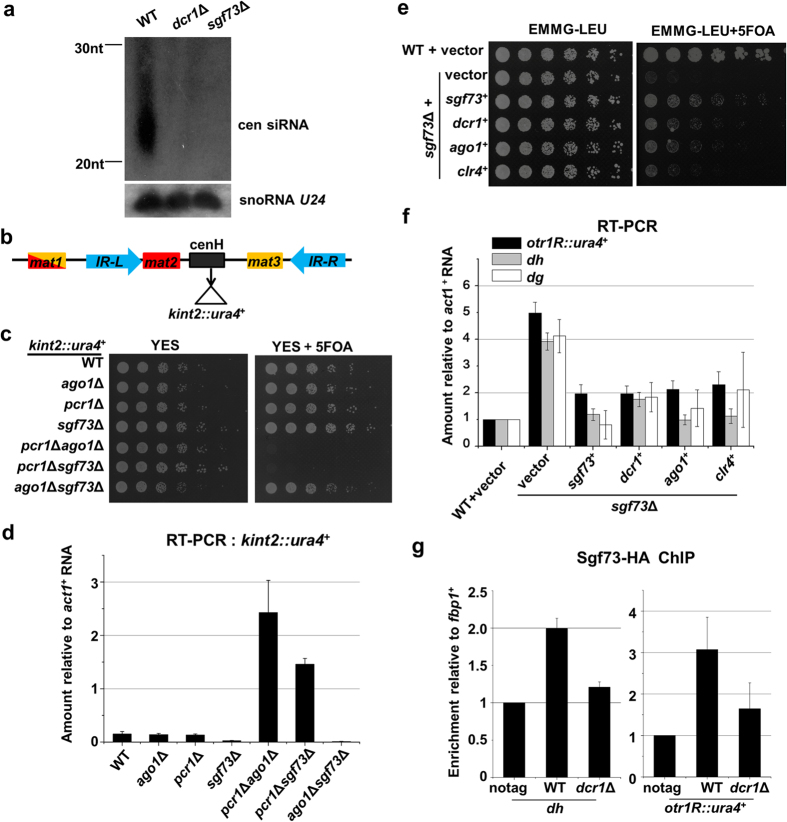
Sgf73 is an essential component of RNAi machinery. (**a**) Northern blot analysis of centromeric siRNAs using probes against *dg* and *dh*. snoRNA U24 was detected as a loading control. (**b**) A schematic representation of mating type region and the position of a marker gene inserted into *K* region (*kint2*::*ura4*^+^). (**c**) Five-fold serial dilution assay to examine the silencing of *kint2*::*ura4*^+^ in *sgf73*Δ cells, and in strains combining deletion of Sgf73 and deletion of a effector in the RNAi or Atf1/Pcr1 pathway. (**d**) RT-PCR analysis of *kint2*::*ura4*^+^ RNA levels from the strains in (**c**). The relative level to a control *act1*^+^ in WT cells was arbitrarily designated as 1 Each column shown in (**d**) and below represents the mean ± s.d. from three biological repeats. (**e**) Fivefold serial dilution assay to examine the silencing of *otr1R*::*ura4*^+^ in *sgf73*Δ cells overexpressing *dcr1*^+^, *ago1*^+^ or *clr4*^+^. *sgf73*Δ cells were transformed with a plasmid overexpressing the indicated gene and transformants were subject to the silencing assay. (**f**) RT-PCR analysis of *dh*, *dg* and *otr1R*::*ura4*^+^ RNA levels relative to a control *act1*^+^ in the transformants in (**e**). (**g**) ChIP analysis of enrichment of Sgf73-3HA at *dh* and *otr1R*::*ura4*^+^ relative to *fbp1*^+^. Relative enrichment in the cells without tagging (no tag) was arbitrarily designated as 1.

In a ChIP assay, Spt7 with a C-terminal triple HA tag (Spt7-3HA) was enriched at *mae2*^+^, a gene targeted by the SAGA complex^35^, but not at the centromeric repeat (*dh*, *dg*) or inserted *ura4*^+^ (Fig. 2d). This result suggests that Spt7, probably in the context of SAGA, is not localized to the pericentromeric heterochromatin region. This is contrast with the enrichment of Sgf73 in heterochromatin shown in Fig. 3 below. Different behaviors between Sgf73 and other subunits of SAGA suggest the role of Sgf73 in the heterochromatin silencing is independent of SAGA. This novel role of Sgf73 might have evolved in fission yeast to cope with different heterochromatin effectors not present in *Saccharomyces cerevisiae*, such as RNAi^37^. On the other hand, a SAGA-independent role of Sgf73 is not an exceptional case in fission yeast. Ataxin-7, a human homologue of yeast Sgf73, is associated with microtubules and enhances the stability of microtubule filaments^38^.

### Sgf73 is an essential component of RNAi machinery

Formation and maintenance of centromeric heterochromatin requires RNAi^3^. As shown in Fig. 1, defective heterochromatin observed in the *sgf73*Δ cells was similar to that in *dcr1*Δ cells, suggesting Sgf73 might be involved in the RNAi pathway. Consistent with this idea, siRNAs corresponding to the pericentromeric repeats (*dg*, *dh*) were substantially decreased in *sgf73*Δ cells, albeit not totally abolished as in *dcr1*Δ cells, while the level of non-coding snoRNA U24 was not affected (Fig. 3a, Supplementary Fig. S2). This suggests Sgf73 is important, but not indispensable for the production of centromeric siRNA.

The involvement of Sgf73 in RNAi machinery was further investigated over the mating type region (*mat2P*/*mat3M*), where RNAi and Atf1/Pcr1 act in parallel pathways to nucleate heterochromatin formation^39^. Deletion of a key component of either pathway, such as Pcr1 or Ago1, did not affect the silencing of a marker *ura4*^+^ gene inserted into the *K* region (*kint2*::*ura4*^+^) (Fig. 3b,c). But disruption of both pathways, as demonstrated by a *pcr1*Δ*ago1*Δ mutant, resulted in the derepression of *ura4*^+^ marker and a severe growth defect on 5FOA (Fig. 3c). *sgf73*Δ and *ago1*Δ *sgf73*Δ cells exhibited normal silencing of *ura4*^+^, but *pcr1*Δ *sgf73*Δ cells were killed on 5FOA due to the derepression of *ura4*^+^ (Fig. 3c). Accordingly, the RNA level of *ura4*^+^ in *pcr1*Δ *sgf73*Δ cells was substantially elevated, as observed in *pcr1*Δ*ago1*Δ cells (Fig. 3d). Overlapping of phenotype of *sgf73*Δ and *ago1*Δ cells in heterochromatin silencing over the mating type region strongly suggests Sgf73 acts in the RNAi pathway.

The relationships between Sgf73 and other RNAi components were validated by a complementation assay. The silencing defect of *otr1R*::*ura4*^+^ in *sgf73*Δ cells was substantially suppressed by overexpressing of *dcr1*^+^, *ago1*^+^ or *clr4*^+^, as shown by the improved growth on 5FOA (Fig. 3e). Accordingly, transcript levels of *ura4*^+^ and centromeric repeats (*dh*, *dg*) decreased in *sgf73*Δ cells overexpressing these factors (Fig. 3f). The result suggests Sgf73 is functionally linked with these key players during RNAi-mediated silencing.

Most of the RNAi components, including RITS, RDRP and dicer, are localized at heterochromatin regions, with exceptions of Arb1 and Arb2^11^,^13^,^15^,^40^. To investigate whether Sgf73 mediates heterochromatin silencing in *cis*, we constructed strains expressing Sgf73 with a C-terminal triple HA tag (Sgf73-3HA). This tag did not affect the gene silencing function of Sgf73 (Supplementary Fig. S3). Compared with a euchromatic locus (*fbp1*^+^), Sgf73-3HA was enriched at the endogenous centromeric repeat (*dh*) and inserted *ura4*^+^ marker (*otr1R::ura4*^+^) (Fig. 3g). Binding of Sgf73-3HA to heterochromatin decreased substantially in *dcr1*Δ cells (Fig. 3g), suggesting siRNA is required for the localization of Sgf73 to the heterochromatin region.

### Sgf73 is required for the assembly and recruitment of RITS complex

**Figure 4:**
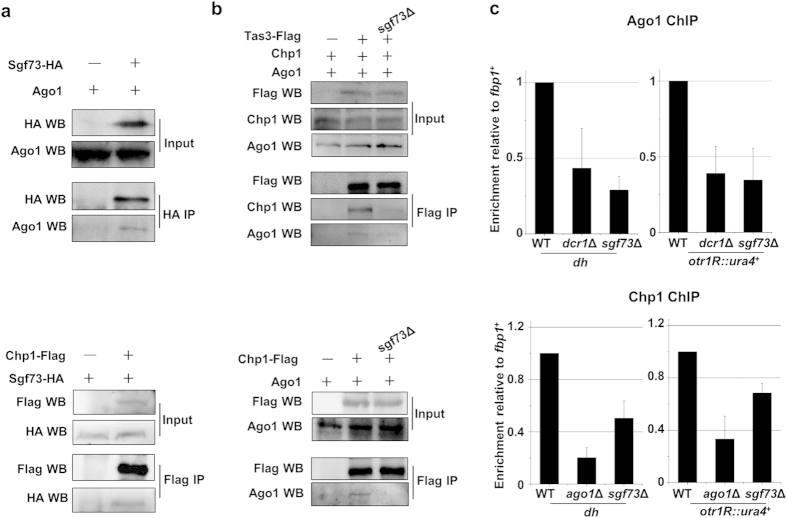
Sgf73 is required for the integrity and recruitment of RITS. (**a**) Co-immunoprecipitation (Co-IP) assay to analyze the physical association between Sgf73 and subunits of RITS. Sgf73-3HA immunoprecipitation (IP) was followed by the Western blot (WB) of Ago1. Chp1-3FLAG IP was followed by the WB of Sgf73-3HA. Input and IP samples were run in the same gel. Cropped blots were shown for clarity. Full-length blots are presented in Supplementary Figs S4 and S5. (**b**) Co-IP assay to analyze the physical association between subunits of RITS in WT and *sgf73*Δ cells. Tas3-3FLAG IP was followed by the WB of Ago1 and Chp1. Chp1-3FLAG IP was followed by the WB of Ago1. Input and IP samples were run in the same gel. Cropped blots were shown for clarity. Full-length blots are presented in Supplementary Figs S6 and S7. (**c**) ChIP analysis of enrichment of Ago1 or Chp1 at *dh* and *otr1R*::*ura4*^+^ relative to *fbp1*^+^. Relative enrichment in the WT cells was arbitrarily designated as 1 Each column represents the mean ± s.d. (*n* = 3) from three biological repeats.

The localization of Sgf73 at the heterochromatin region suggests Sgf73 is physically associated with other RNAi components. As shown in Fig. 4a, Ago1 and Chp1, subunits of RITS complex, were co-purified with Sgf73-3HA in a co-immunoprecipitation assay. This suggests that Sgf73 is associated with RITS. Since Sgf73 was not among the peptides identified by tandem affinity purification using Chp1 as bait^15^, Sgf73 is unlikely to be a fundamental subunit of the RITS complex. Instead, Sgf73 is perhaps a peripheral component of RITS. Notably, the interactions among the subunits of the RITS, including that between Tas3 and Chp1, between Chp1 and Ago1, were severely impaired by the deletion of *sgf73*^+^ (Fig. 4b). Ago1-Tas3 interaction was also disrupted, but to a much lesser extent, in *sgf73*Δ cells (Fig. 4b). Sgf73 is the first known factor that regulates the integrity of RITS. In contrast, integrity of RITS is not dependent on the production of siRNA or H3K9 methylation, as the Tas3-Ago1-Chp1 composition of RITS is not affected by the deletion of *dcr1*^+^or *clr4*^+^ 15. A previous study indicates that the physical interaction between Ago1 and Tas3 is required for the recruitment of Ago1 to centromeres for efficient *de novo* establishment of centromeric heterochromatin^41^. Accordingly, the enrichments of Ago1 at the centromeric repeat (*dh*) and inserted *ura4*^+^ marker decreased substantially in *sgf73*Δ cells (Fig. 4c). The enrichment of Chp1 at both loci also decreased, but to a lesser extent comparing to those of Ago1, which might be explained by the tethering of Chp1 with residual H3K9me2 in *sgf73*Δ cells^17^. The results suggest that Sgf73 is critical for the recruitment of RITS to the centromeric heterochromatin.

## Conclusions

In this study, we revealed an essential role of Sgf73 in RNAi-mediated heterochromatin silencing. Sgf73 is physically associated with RITS and is required for the integrity of RITS. Given the fact that the localizations of Ago1 and Chp1 at heterochromatin were both impaired in *sgf73*Δ cells, it suggests that RITS need to be fully assembled before it is recruited to the centromeric region to initiate heterochromatin silencing. However, the structural integrity of RITS is not required afterwards to maintain the localization of individual subunits at the centromeric region, as demonstrated by the phenotype of a Tas3 mutant which failed to interact with Ago1^41^. The recruitment of Sgf73 to the centromeric region is probably mediated through the associations with RITS, as the localization of Sgf73 relies on Dcr1 that produces siRNAs to guide RITS.

This study also extends a growing list of the moonlighting functions of SAGA subunits. Besides Sgf73, Spt20, a structural subunit of SAGA, regulates septin ring assembly through physical interactions with septins^42^. p38IP, a human homologue of Spt20, interacts with mammalian (m)Atg9 (ATG9A) and inhibits the trafficking of mAtg9^43^. Tra1, the largest subunit of SAGA, was identified as a subunit of a novel complex called ASTRA in fission yeast^44^. Divergently evolved functions of SAGA subunits reflect the capacity of a subunit in a large complex to acquire additional functions to adapt to new environments.

## Methods

### Yeast strains and plasmids

All the strains used in this study are listed in Supplementary Table S1. Gene deletion and tagging were performed by homologous recombination using a plasmid-based method^45^. Cells were grown in yeast extract medium with supplements (YES) or Edinburgh minimal glutamate medium minus leucine (EMMG-Leu) medium^46^. ORF of *sgf73*^+^, *ago1*^+^, *dcr1*^+^ or *clr4*^+^ was cloned into pRep41 vector for overexpressing^47^.

### Fivefold serial dilution assay

Exponentially growing cells were collected and adjusted to an A_600_ of 1.0. Samples were diluted by fivefold for five times. 5 μl dilutions were spotted onto YES or EMMG-Leu medium supplemented with 5FOA (YY12210, Yuanye Biotechnology, Shanghai, China, 1 g/Liter) as indicated. Plates were incubated for 2 or 3d at 32 °C before imaging.

### RT-PCR

1 × 10^8^ exponentially growing cells were harvested. Total RNA were extracted using the RiboPure Yeast (AM1926, Life Technologies, Carlsbad, CA, USA) and reverse transcribed into cDNA by using PrimeScript RT (RR037A, Takara, Dalian, China). qPCR was performed using SYBR Premix Ex TaqII (RR820A, Takara) in a LightCycler 480 II Real-Time PCR System (Roche Applied Science, Penzberg, Upper Bavaria, Germany). Primers used are listed in Supplementary Table S2.

### ChIP

3 × 10^8^ exponentially growing cells were fixed with 1% formaldehyde for 25 min at 30 °C. After quenching by 250 mM glycine, cells were harvested and washed with Buffer 1 (1 M Tris-HCl (pH 8.0), 167 mM NaCl, 1.2 mM EDTA, 1% TritonX-100, 0.1% Na-deoxycholate). Cells were resuspended in Buffer 1 supplemented with protease inhibitors cocktail (05892970001, Roche Applied Science) and homogenized with a bead-beater (FastPrep-24, MP, California, USA) by glass beads. The cell extract was sonicated for 15 min with a sonicator (Sonics & Materials, Connecticut, USA) and centrifuged. Supernatant was incubated with anti-HA (M20003L, Abmart, Shanghai, China), anti-H3K9me2 (07-441, Millipore, Massachusetts, USA), anti-Ago1 (ab18190, Abcam, Cambs, UK, Abcam), anti-RNA polymerase II 8WG16 (MMS-126R, Covance, New Jersey, USA), anti-FLAG (F1804-200UG, Sigma-Aldrich, St Louis, MO, USA) or anti-Chp1 (ab18181, Abcam) antibody for 4 hour. Samples were subjected to purification by using an EZ-Magna ChIP A Kit (17-408, Millipore). Eluted DNA was subjected to qPCR as described above. Primers used are listed in Supplementary Table S2.

### Co-immunoprecipitation

~5 × 10^10^ exponentially growing cells were harvested. Cells were washed and then resuspended in Buffer 1 supplemented with protease inhibitors as described above. Cells were broken by a high-pressure cell homogenizer (JN-02C, JNBIO, Guangzhou, China) and supernatant were collected after centrifugation. Supernatant was incubated with anti-FLAG M2 Magnetic beads (M8823, Sigma-Aldrich), or with anti-HA antibody and then protein A/G PLUS-agarose beads (sc-2003, Santa Cruz, Dallas, TX, USA) for 6h. Bead-conjugated complexes were washed with Buffer 1, boiled in SDS-gel loading buffer and subjected to Western blot.

### Northern blot

Small RNA fractions were prepared from exponentially growing cells using mirVana miRNA Isolation kit (AM1560, Life Technologies). Small RNA was resuspended in 50% formamide and separated on a 15% urea-denaturing poly-acrylamide gel. Samples on the gel were blotted onto a Hybond-N membrane (RPN303N, GE Healthcare, Piscataway, NJ, USA) using a semi-dry electrophoretic transfer cell (170-3912, Bio-Rad, California, USA). ^32^P labeled oligonucleotide probes complimentary to *dh* and *dg* were generated by PCR. Probe against snoU24 was generated by end-labelling. Probes were hybridized to the membrane overnight at 38 °C in a rotating oven. The membrane was then washed twice and exposed to an X-ray film for 3 d at −80 °C. Primers and oligos used are listed in Supplementary Table S2.

## Additional Information

**How to cite this article**: Deng, X. *et al.* Sgf73, a subunit of SAGA complex, is required for the assembly of RITS complex in fission yeast. *Sci. Rep.* **5**, 14707; doi: 10.1038/srep14707 (2015).

## Supplementary Material

Supplementary Information
