# Supplementary material for: Sgf73, a subunit of SAGA complex, is required for the assembly of RITS complex in fission yeast
Source: Sci Rep. 2015 Oct 7;5:14707. doi: 10.1038/srep14707 (PMC4595766; doi:10.1038/srep14707)
Supplement: Supplementary Information [file srep14707-s1.doc]

**Supplementary Information**

**Sgf73, a subunit of SAGA complex, is required for the assembly of RITS complex in fission yeast**

Xiaolong Deng^1,2^, Huan Zhou^1,2^, Guiping Zhang^3^, Wenchao Wang^1,2^, Langyong Mao^1,2^, Xing Zhou^1,2^, Yao Yu^1,2^*, Hong Lu^1,3,4^*

^1^ State Key Laboratory of Genetic Engineering, School of Life Sciences, Fudan University

^2^ Shanghai Engineering Research Center Of Industrial Microorganisms, Shanghai, China, 200438

^3^ Shanghai Center for Plant Stress Biology, Shanghai Institutes for Biological Sciences, Chinese Academy of Sciences, Shanghai, China, 201602

^4^ Shanghai Collaborative Innovation Center for Biomanufacturing Technology, Shanghai, China, 200237

*Corresponding authors. [yaoyu@fudan.edu.cn](mailto:yaoyu@fudan.edu.cn) (YY), [honglu0211@yahoo.com](mailto:honglu0211@yahoo.com) (HL)


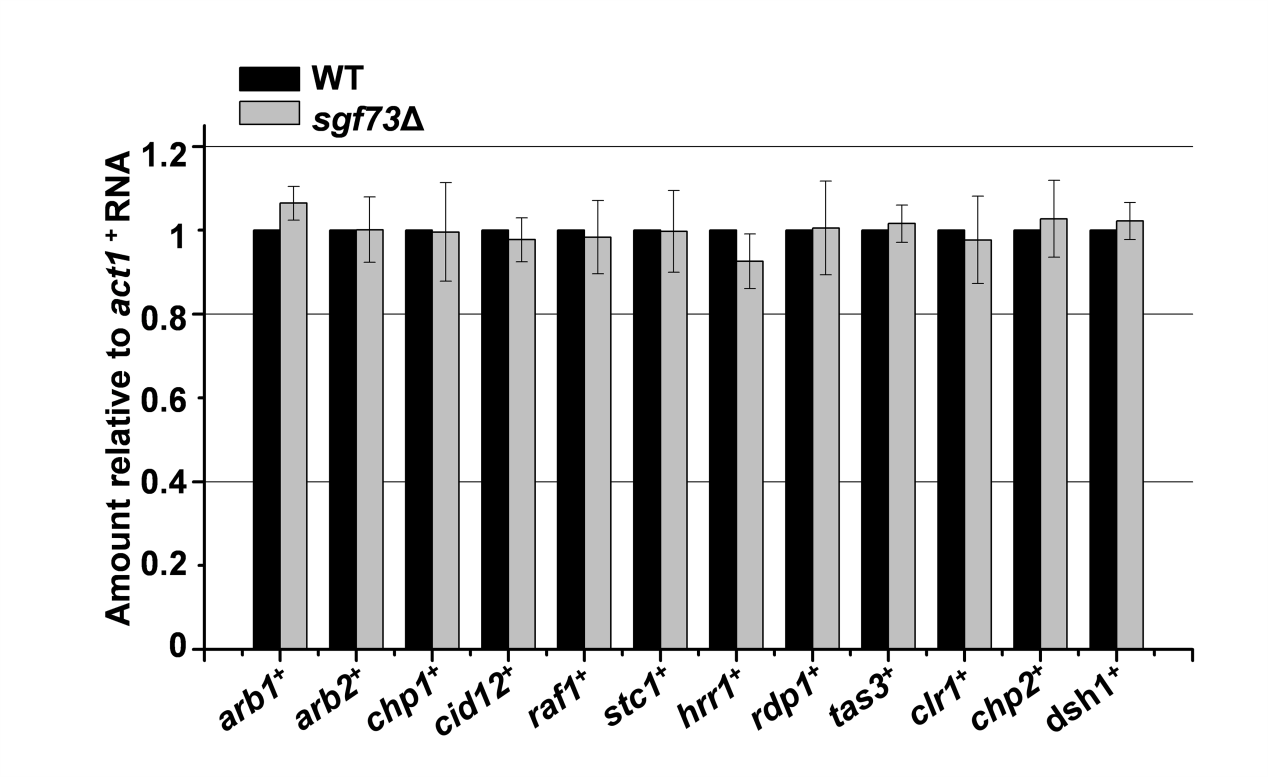


**Supplementary Figure S1. Sgf73 is not required for the transcription of essential silencing factors.**

RT-PCR analysis of RNA levels of representative silencing factors in WT and *sgf73*Δ cells. The relative level to a control *act1*^+^ in WT cells was arbitrarily designated as 1. Each column represents the mean±s.d. from three biological repeats.


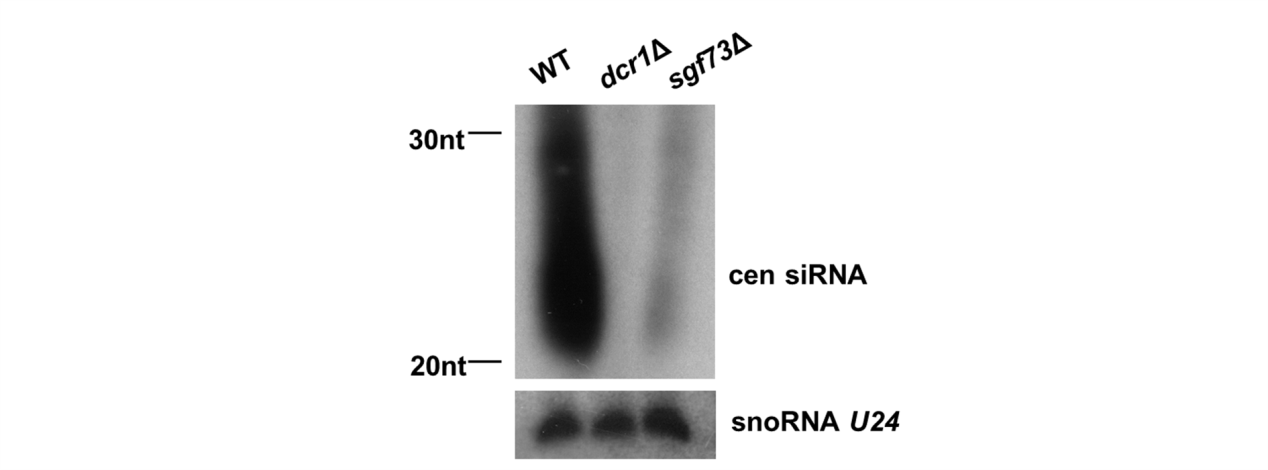


**Supplementary Figure S2. Production of centromeric siRNAs was severely impaired, but not totally abolished in *sgf73*Δ cells.**

Northern blot analysis of centromeric siRNAs using probes against *dg* and *dh* by long exposure. snoRNA U24 was detected as a loading control.


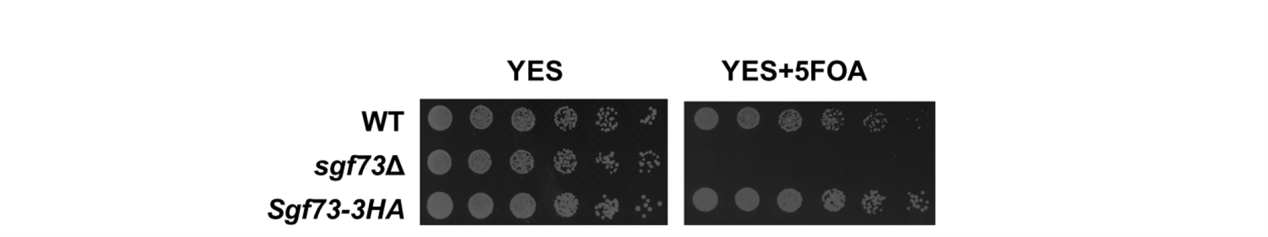


**Supplementary Figure S3. Sgf73-3HA strain displayed normal silencing at pericentromeric region.**

Fivefold serial dilution assay to examine the silencing of *otr1R*::*ura4*^+^ in strain expressing Sgf73 with a C terminal triple HA tag (Sgf73-3HA). Wild-type (WT) cells without tagging and *sgf73*Δ cells were assayed as controls.


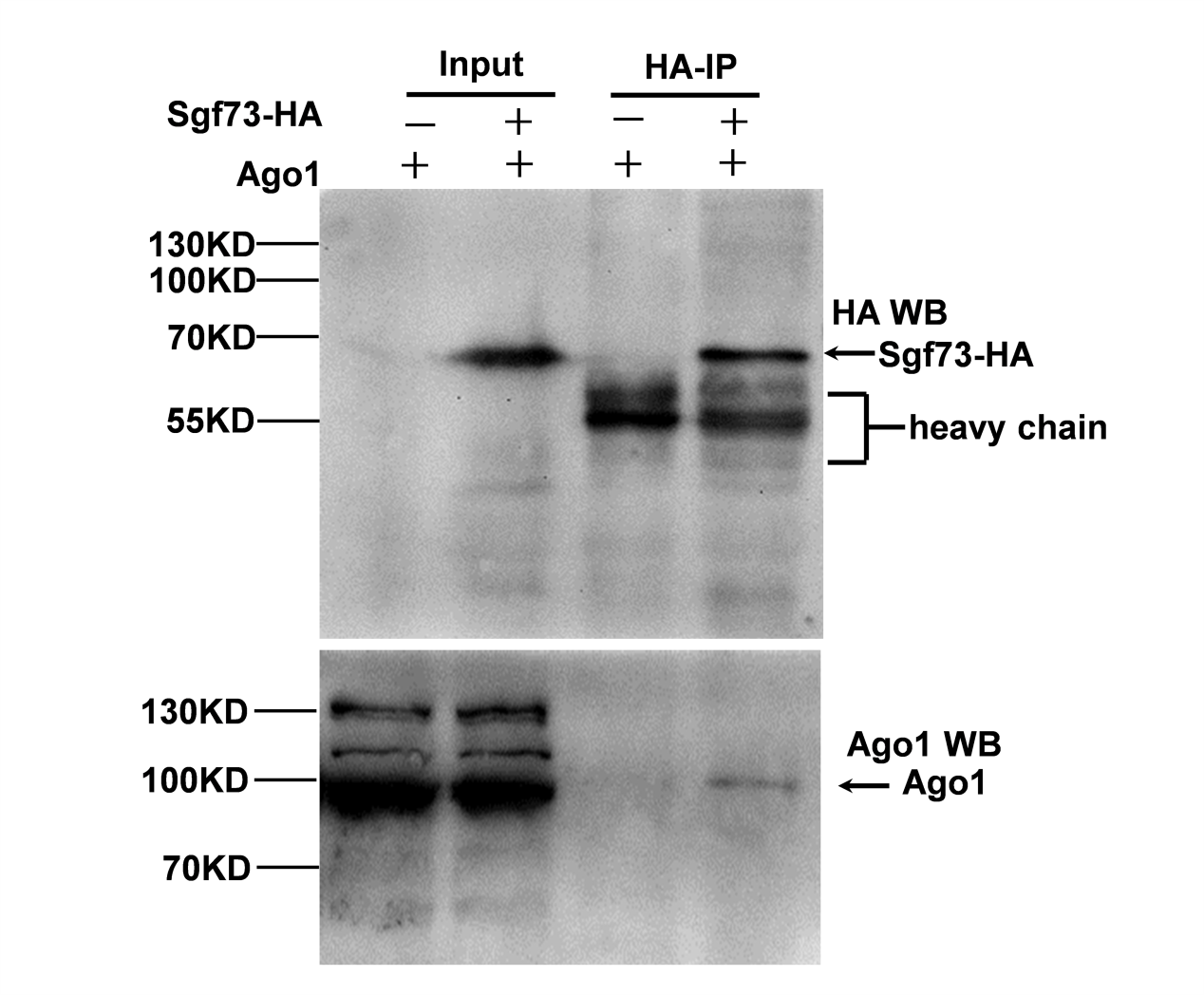


**Supplementary Figure S4. Full-length blots of a Co-IP assay in Fig. 4a (upper panel).**

Sgf73-3HA immunoprecipitation (IP) was followed by the Western blot (WB) of Ago1.


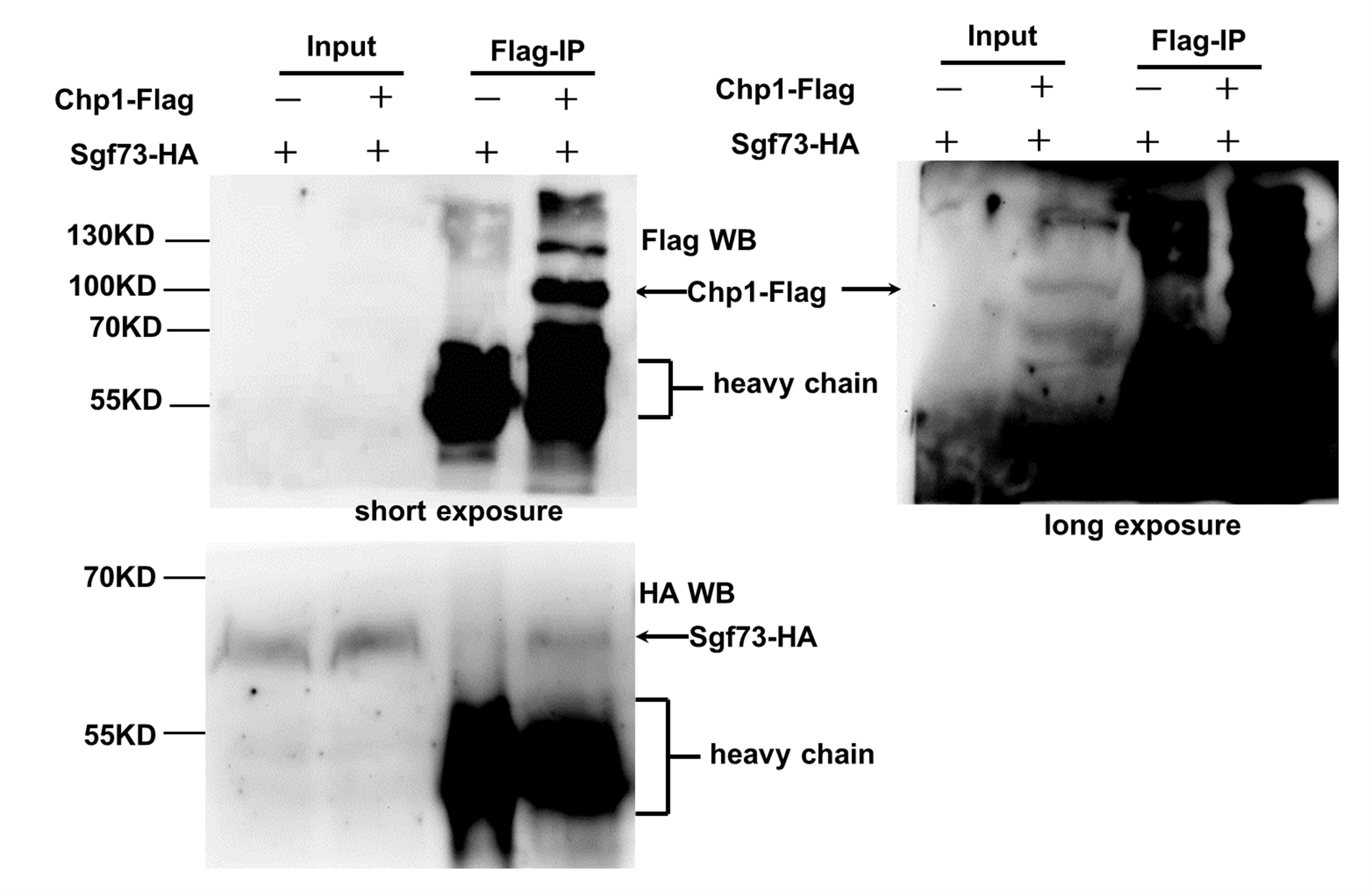


**Supplementary Figure S5. Full-length blots of a Co-IP assay in Fig. 4a (lower panel).**

Chp1-FLAG IP was followed by the WB of Sgf73-3HA. Because the level of Chp1-FLAG in cell lysate was low, input of Chp1-FLAG was cropped from a blot with long exposure, while IP of Chp1-FLAG was cropped from the same blot with short exposure.


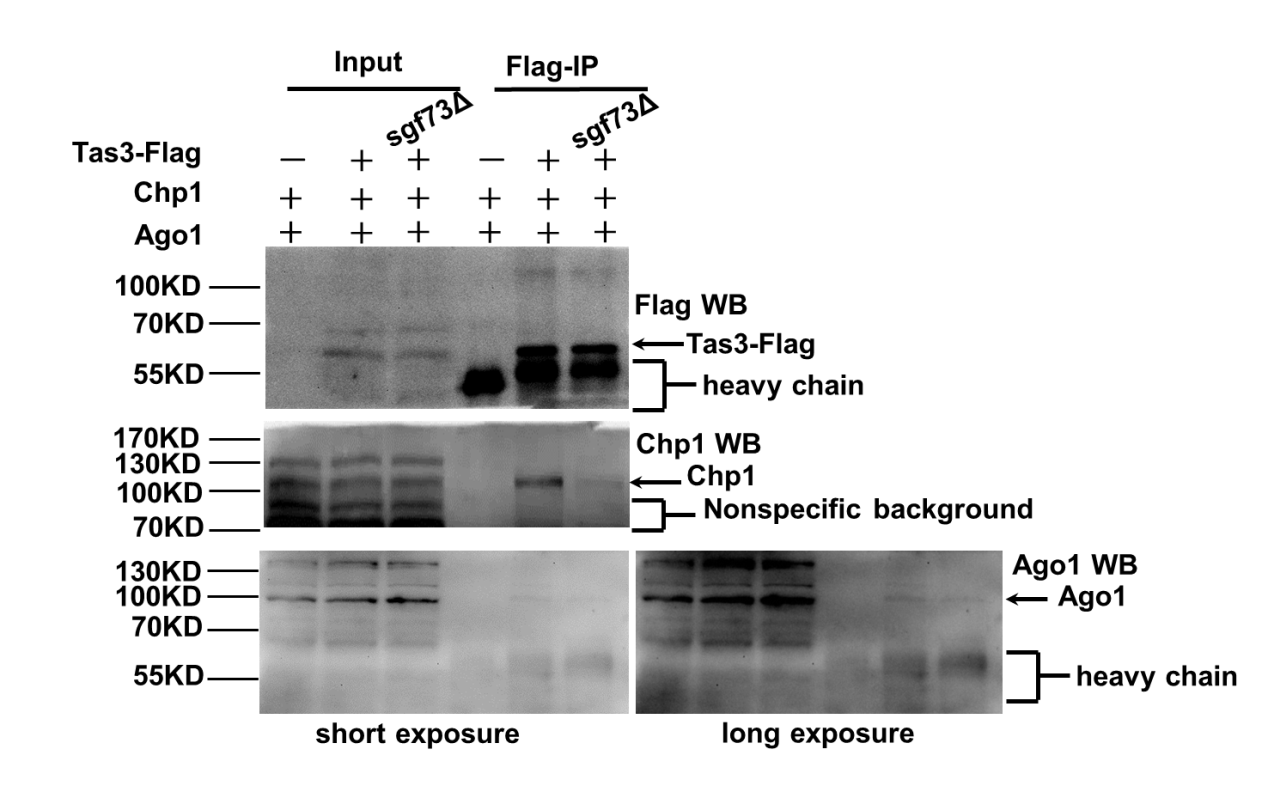


**Supplementary Figure S6. Full-length blots of a Co-IP in Fig. 4b (upper panel).**

Tas3-3FLAG IP was followed by the WB of Ago1 and Chp1. Because the level of Ago1 after IP was low, IP of Ago1 was cropped from a blot with long exposure, while input of Ago1 was cropped from the same blot with short exposure.


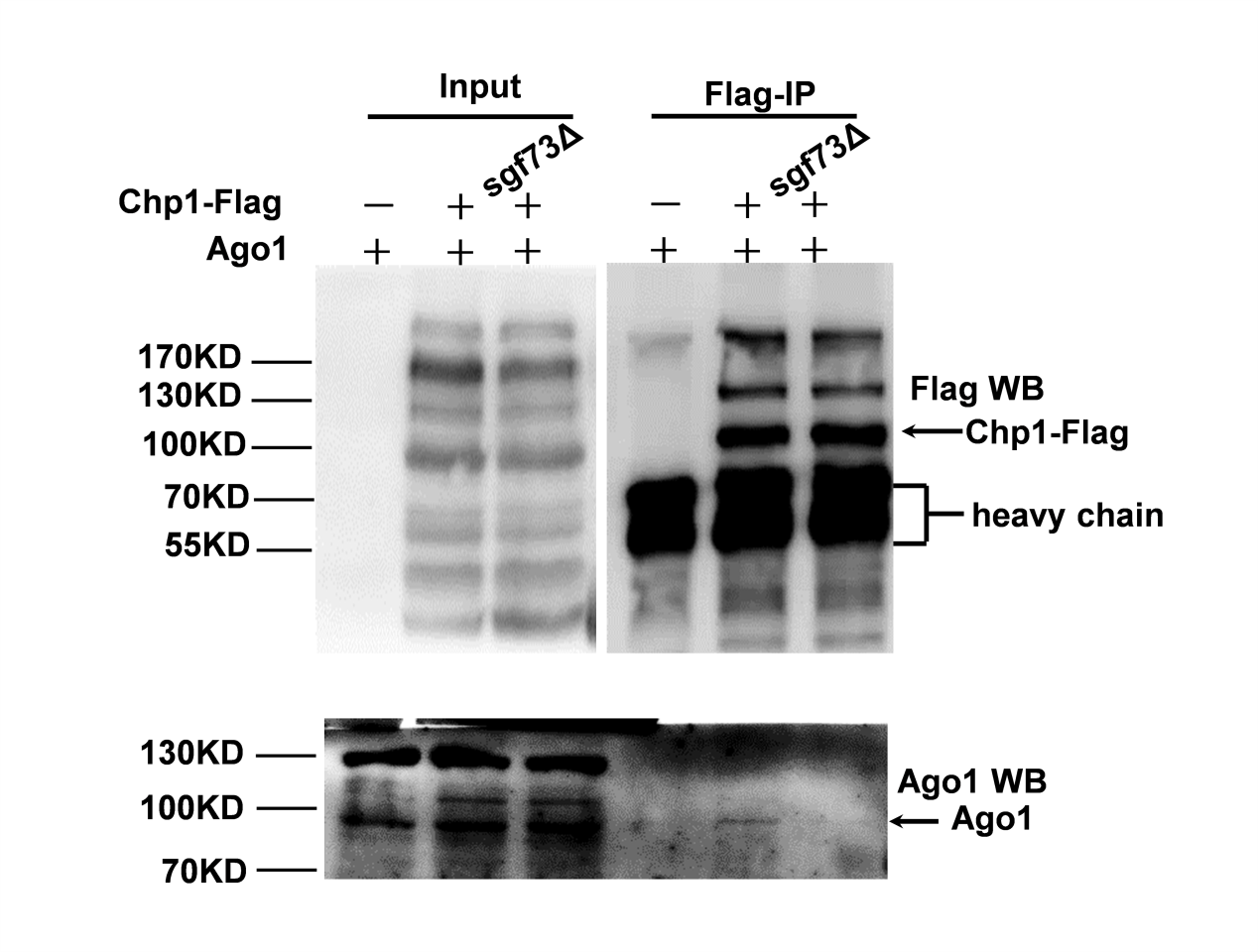


**Supplementary Figure S7. Full-length blots of a Co-IP assay in Fig. 4b (lower panel).**

Chp1-FLAG IP was followed by the WB of Ago1. Because the level of Chp1-FLAG in cell lysate was low, input of Chp1-FLAG was cropped from a blot with long exposure, while IP of Chp1-FLAG was cropped from a different blot with short exposure. The position of Chp1-FLAG in different blots was identified by molecular markers.

**Supplementary Table S**1. List of strains used in this study.

| **Strain** | **Genotype** | **Source** |
| --- | --- | --- |
| FY648 | *h^+^,leu1-32,ade6-m210,ura4-DS/E,otr1R(SphI)::ura4^+^* | Robin Allshire (1) |
| LHP305 | *h^+^,leu1-32,ade6-m210,ura4-DS/E,otr1R(SphI)::ura4^+^,sgf73*Δ*::kanMX6* | constructed in this study |
| LHP306 | *h^+^,leu1-32,ade6-m210,ura4-DS/E,otr1R(SphI)::ura4^+^,dcr1*Δ*::hphMX6* | constructed in this study |
| LHP307 | *h^+^,leu1-32,ade6-m210,ura4-DS/E,otr1R(SphI)::ura4^+^,ubp8*Δ*::hphMMX6* | constructed in this study |
| LHP308 | *h^+^,leu1-32,ade6-m210,ura4-DS/E,otr1R(SphI)::ura4^+^,gcn5*Δ*::hphMMX6* | constructed in this study |
| LHP309 | *h^+^,leu1-32,ade6-m210,ura4-DS/E,otr1R(SphI)::ura4^+^,sgf29*Δ*::hphMMX6* | constructed in this study |
| LHP310 | *h^+^,leu1-32,ade6-m210,ura4-DS/E,otr1R(SphI)::ura4^+^,spt7*Δ*::hphMMX6* | constructed in this study |
| LHP311 | *h^+^,leu1-32,ade6-m210,ura4-DS/E,otr1R(SphI)::ura4^+^,sus1*Δ*::hphMMX6* | constructed in this study |
| LHP312 | *h^+^,leu1-32,ade6-m210,ura4-DS/E,otr1R(SphI)::ura4^+^,sgf11*Δ*::hphMMX6* | constructed in this study |
| LHP313 | *h^+^,leu1-32,ade6-m210,ura4-DS/E,otr1R(SphI)::ura4^+^,sgf73-3HA::kanMX6* | constructed in this study |
| LHP314 | *h^+^,leu1-32,ade6-m210,ura4-DS/E,otr1R(SphI)::ura4^+^,sgf73-3HA::kanMX6,dcr1*Δ*::hphMX6* | constructed in this study |
| LHP315 | *h^+^,leu1-32,ade6-m210,ura4-DS/E,otr1R(SphI)::ura4^+^,dcr1-3Flag::hphMX6* | constructed in this study |
| LHP316 | *h^+^,leu1-32,ade6-m210,ura4-DS/E,otr1R(SphI)::ura4^+^,dcr1-3Flag::hphMX6,sgf73*Δ*::kanMX6* | constructed in this study |
| LHP317 | *h^+^,leu1-32,ade6-m210,ura4-DS/E,otr1R(SphI)::ura4^+^,spt7-3HA::kanMX6* | constructed in this study |
| LHP318 | *h^+^,leu1-32,ade6-m210,ura4-DS/E,otr1R(SphI)::ura4^+^,stc1-Flag::hphMX6* | constructed in this study |
| LHP319 | *h^+^,leu1-32,ade6-m210,ura4-DS/E,otr1R(SphI)::ura4^+^,stc1-Flag::hphMX6,sgf73*Δ*::kanMX6* | constructed in this study |
| LHP320 | *h^+^,leu1-32,ade6-m210,ura4-DS/E,otr1R(SphI)::ura4^+^, tas3-3Flag::hphMX6* | constructed in this study |
| LHP321 | *h^+^,leu1-32,ade6-m210,ura4-DS/E,otr1R(SphI)::ura4^+^, tas3-3Flag::hphMX6,sgf73Δ::kanMX6* | constructed in this study |
| LHP328 | *h+,leu1-32,ade6-m210,ura4-DS/E,otr1R(SphI)::ura4+,chp1-3Flag::hphMX6,sgf73-HA::kanMX6* | constructed in this study |
| SPJ83 | *h^90^,his2, leu1-32, ura4-DS/E, ade6-m210, kint2::ura4^+^* | Shiv Grewal (2) |
| LHP322 | *h^90^,his2, leu1-32, ura4-DS/E, ade6-m210, kint2::ura4^+^,ago1*Δ*::kanMX6* | constructed in this study |
| LHP323 | *h^90^,his2, leu1-32, ura4-DS/E, ade6-m210, kint2::ura4^+^,sgf73*Δ*::hphMMX6* | constructed in this study |
| LHP324 | *h^90^,his2, leu1-32, ura4-DS/E, ade6-m210, kint2::ura4^+^,pcr1*Δ*::hphMMX6* | constructed in this study |
| LHP325 | *h^90^,his2, leu1-32, ura4-DS/E, ade6-m210, kint2::ura4^+^,ago1*Δ*::kanMX6,sgf73*Δ*::hphMX6* | constructed in this study |
| LHP326 | *h^90^,his2, leu1-32, ura4-DS/E, ade6-m210, kint2::ura4^+^,pcr1*Δ*::kanMX6,sgf73*Δ*::hphMX6* | constructed in this study |
| LHP327 | *h^90^,his2, leu1-32, ura4-DS/E, ade6-m210, kint2::ura4^+^,pcr1*Δ*::kanMX6,ago1*Δ*::hphMX6* | constructed in this study |

Reference:

[1] Allshire RC, Nimmo ER, Ekwall K, Javerzat JP, Cranston G (1995) Mutations derepressing silent centromeric domains in fission yeast disrupt chromosome segregation. *Genes Dev* **9:** 218-233

[2] Jia S, Noma K, Grewal SI (2004) RNAi-independent heterochromatin nucleation by the stress-activated ATF/CREB family proteins. *Science* **304:** 1971-1976

**Supplementary Table S**2. Primers used in Real-Time PCR and siRNA analysis

| **Primer name** | **Sequence** | **Used for** |
| --- | --- | --- |
| *act1+* forward | AACCCTCAGCTTTGGGTCTT | RT-PCR |
| *act1+* reverse | TTTGCATACGATCGGCAATA | RT-PCR |
| *otr dh* forward | ATGAAATCGTTTACCGCTTCTCC | ChIP, RT-PCR |
| otr *dh* reverse | TTGAAGATGGCGTATGTAGTGCT | ChIP, RT-PCR |
| otr *dg* forward | CCATCACCACTTTCATCTCC | ChIP, RT-PCR |
| otr *dg* reverse | CAGGATACCTAGACGCACAA | ChIP, RT-PCR |
| *otrR::ura4*^+^ forward | GAATGGTTTGAGAAGCATACC | ChIP, RT-PCR |
| *otrR::ura4*^+^ reverse | GAGTACGATATTGCTGTCCC | ChIP, RT-PCR |
| *fbp1*^+^ forward | GTCGAACGGATGCTGCAAAC | ChIP |
| *fbp1*^+^ reverse | GGTACCTACACTAACACCGG | ChIP |
| *mae2*^+^ forward | CCTGGCAACACCCGAAGTTAT | ChIP |
| *mae2*^+^ reverse | CGTTCGGTTATAGCAGTAGTTGTCAC | ChIP |
| *ago1*^+^ forward | GCTCCTATTAGATCTTGGGC | RT-PCR |
| *ago1*^+^ reverse | TAGTCAGGAGGAGCATTACC | RT-PCR |
| *dcr1*^+^ forward | CTATATTCTCCCAGTCTCTA | RT-PCR |
| *dcr1*^+^ reverse | TGGTACTCTTGAGTATCAGG | RT-PCR |
| *clr4*^+^ forward | GAGAGAGCTATTTCGTAAGA | RT-PCR |
| *clr4*^+^ reverse | CCCCTGTGCATCATAGGCAA | RT-PCR |
| *rik*^+^ forward | GTGTGTACCATCTGCATGAT | RT-PCR |
| *rik1*^+^ reverse | CGATAACCAAGTCTCCATCA | RT-PCR |
| *swi6*^+^ forward | CCAGTGATAATACATGGAGT | RT-PCR |
| *swi6*^+^ reverse | GAAGGTGGGCCGTTCTCATT | RT-PCR |
| *clr3*^+^ forward | GCTGCCTATGCTCATATGAC | RT-PCR |
| *clr3*^+^ reverse | TTAGGATTTGCGTCGAAGTG | RT-PCR |
| *cen* probe1 forward | AGTCAACTGAACAACGCATCTAC | Northern |
| *cen* probe1 reverse | AACTCCTGCTTATCGTCTTCTTT | Northern |
| *cen* probe2 forward | ATCTGCCATCACTTTATTTCTCC | Northern |
| *cen* probe2 reverse | TCAACCTTCCGACGCAAATCACC | Northern |
| *cen* probe3 forward | AATATGCTGCGGTTCACCCTTAA | Northern |
| *cen* probe3 reverse | TAGCCATTTGCTTAACTTACTGTCTCA | Northern |
| *cen* probe4 forward | TCATCAGCCTCTCTCTATATCTCTA | Northern |
| *cen* probe4 reverse | GACAGAATGGATGGATATTGACAG | Northern |
| *snoRNA U24* probe | GATTTGTTTTGTCTCATCGAGCC | Northern |
